# A Comparison of Fully-Coupled 3D In-Stent Restenosis Simulations to *In-vivo* Data

**DOI:** 10.3389/fphys.2017.00284

**Published:** 2017-05-23

**Authors:** Pavel S. Zun, Tatiana Anikina, Andrew Svitenkov, Alfons G. Hoekstra

**Affiliations:** ^1^Saint Petersburg State University of Information Technologies, Mechanics and Optics (ITMO) UniversitySt. Petersburg, Russia; ^2^Computational Science Lab, Faculty of Science, Institute for Informatics, University of AmsterdamAmsterdam, Netherlands

**Keywords:** multiscale modelling, in-stent restenosis, hemodynamics, agent-based modelling, coronary vessel stenosis

## Abstract

We describe our fully-coupled 3D multiscale model of in-stent restenosis, with blood flow simulations coupled to smooth muscle cell proliferation, and report results of numerical simulations performed with this model. This novel model is based on several previously reported 2D models. We study the effects of various parameters on the process of restenosis and compare with *in vivo* porcine data where we observe good qualitative agreement. We study the effects of stent deployment depth (and related injury score), reendothelization speed, and simulate the effect of stent width. Also we demonstrate that we are now capable to simulate restenosis in real-sized (18 mm long, 2.8 mm wide) vessel geometries.

## Introduction

Cardiovascular diseases are one of the leading causes of death in industrialized countries, and because of that many studies focus on preventing and treating these diseases. One important type of cardiovascular disease is coronary artery disease (CAD) and specifically the abnormal narrowing, or stenosis, of coronary arteries. This kind of narrowing restricts the flow of blood to the heart muscle, often leading to ischemia and myocardial infarction (MI). This condition is routinely corrected by balloon angioplasty. Currently, during balloon angioplasty, a metal mesh, or stent, is usually placed inside the vessel to keep it open after the vessel lumen is enlarged by the inflating balloon (Iqbal et al., 2013).

However, this procedure causes damage to the vessel wall, both by the balloon and by the stent struts that are pressed deep into the intimal, medial, and sometimes even adventitial layer of the arterial wall. This damage causes a healing response, which consists of inflammation, endothelial and smooth muscle cell proliferation, and other processes. Also, the exposed stent struts in the lumen provoke thrombus formation (Jukema et al., 2012a,b).

Because of this, two widespread adverse effects of stenting are restenosis and stent thrombosis. Stent thrombosis happens when too many thrombocytes aggregate on the stent struts and block the artery. This can occur days or even hours after stenting, although in some cases late stent thrombosis happens months or even years after the procedure. Thrombosis is a very dangerous condition and can lead to a potentially fatal MI unless immediately treated. Restenosis is a less acute complication. It was very common during the era of bare metal stents (BMS). Now with drug-eluting stents (DES) it happens more rarely, although it is still relatively widespread (Chieffo et al., 2009) and can lead to increased morbidity, ischemia, and increases the risk of late thrombosis.

Restenosis is caused by a maladaptive healing response of the vessel wall. Immediately after angioplasty, most of the endothelium is damaged and dysfunctional, and smooth muscle cells (SMCs) at the injury site change their phenotype from contractile to synthetic and start proliferating (Evans et al., 2008; Jukema et al., 2012a; Tahir et al., 2013, 2014). Normally, the SMCs proliferate enough to cover the stent struts in neointima, and after that the endothelium recovers its function and suppresses SMC growth, causing them to switch back to their contractile phenotype. However, the healing is not always perfect. Sometimes, especially if the stent is covered in anti-proliferative drugs, the growth stops too early, leaving some struts uncovered, greatly increasing the risk of late thrombus formation in the stent (Lagerqvist et al., 2009). In other cases, the growth is too intensive, and it causes a repeat narrowing of the lumen, or restenosis. This narrowing restricts blood flow to the distal tissue, causing ischemia and possibly tissue death. Also, it adds a risk that a thrombus originating in systemic veins can block the stented segment completely, causing acute MI (Kolandaivelu et al., 2011).

The probable causes and dynamics of restenosis, as well as possible ways to reduce it, were studied in many clinical trials and animal experiments (see e.g., Jukema et al., 2012a,b; Meier et al., 2012; De Caterina et al., 2013; Goel et al., 2013; Iqbal et al., 2013; Giacoppo et al., 2015). Also, there exist several analytical and computational models of this process (Prendergast et al., 2003; Boyle et al., 2011, 2013; Keller et al., 2014; Nolan et al., 2014; Zahedmanesh et al., 2014), including those developed earlier by our group (Evans et al., 2008; Tahir et al., 2011, 2015; Amatruda et al., 2014). For example, Zahedmanesh et al. (2014) use a two-dimensional (2D) finite element method (FEM) model of stent deployment coupled with an (also 2D) agent-based model of SMC proliferation and extracellular matrix (ECM) generation. Boyle et al. (2013) couple a 3D FEM model of stent expansion to a lattice-based cell growth model. To the best or our knowledge, our model is unique in that it captures the neointima formation *also* as a function of the wall shear stresses due to blood flow, which is explicitly computed. Moreover, we take reendothelization into account, and this is exactly where the bloodflow is coupled to the dynamics of the neointima formation (Tahir et al., 2013). So far we mainly simulated ISR using a two-dimensional (2D) version of the model. In this paper we report on first results obtained by using a computational three-dimensional (3D) multiscale model of in-stent restenosis (ISR3D) in which SMC proliferation and re-endothelialization are fully coupled to blood flow simulations. We compare these results to *in-vivo* experimental data.

We model the restenotic response in a straight cylindrical porcine coronary vessel with no atherosclerotic lesions, which has been injured by an over-inflated balloon, and compare the results with *in-vivo* porcine data obtained in a similar experimental setup (Gunn et al., 2002; Morton et al., 2004; Tahir et al., 2011). We consider cases of different deployment depths which cause different degrees of injury to the artery, and also model scenarios of early and late endothelial recovery.

## Methods and model description

In this section we describe the computational model we use in this study, which is based on our earlier studies relying on a two-dimensional formulation (Tahir et al., 2011, 2013, 2014, 2015; Amatruda et al., 2014). As was mentioned earlier, in-stent restenosis is a complex process that depends on a multitude of biological and physical processes that act on a range of spatial and temporal scales. A single-scale model that accounts for all these processes on the smallest required spatio-temporal resolution would require unreasonable computation time. So, to account for these processes, a multi-scale modelling approach is used (Evans et al., 2008; Caiazzo et al., 2011; Groen et al., 2013; Chopard et al., 2014; Hoekstra et al., 2016). In our model we explicitly model the blood flow, the neointima, the internal elastic lamina (IEL), the media, and the external elastic lamina (EEL). The endothelium is modelled implicitly and the adventitia is assumed to hold the EEL in a set position after the stent is deployed. Note that with respect to the original two-dimensional model (Caiazzo et al., 2011) we added the EEL, and the reendothelialization (Tahir et al., 2013).

**Figure 1:**
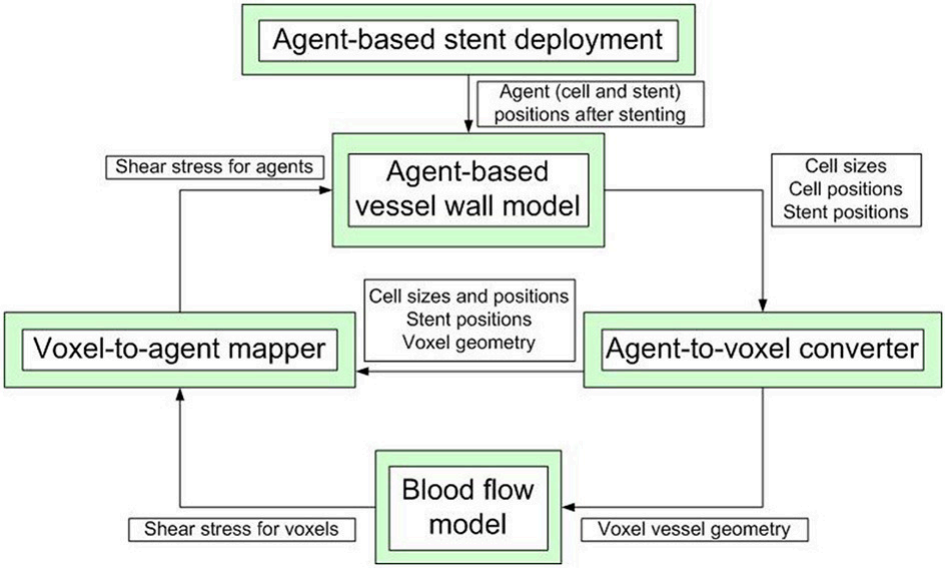
**A simplified scheme of communication between the submodels**. The simulation starts with stent deployment, which provides the initial configuration for growth and proceeds for a pre-set number of agent-based model iterations.

Our model is composed of several single-scale submodels, each with its own spatial and temporal scale, that are used to model blood flow inside the vessel, stent deployment and cell growth and proliferaton. These submodels communicate relevant values to each other during the simulation. A simplified scheme of inter-model communication is shown in Figure 1. ISR3D contains submodels for physical interaction of smooth muscle cells, stent struts, and other components of the vessel wall, biological growth and proliferation of SMCs, and blood flow through the vessel. Unless otherwise noted, all simulations described below were performed for a 8 mm long BiodivYsio stent segment deployed inside a 2.8 mm wide cylindrical vessel. We are going to describe the submodels in some detail in the next paragraphs.

We model all individual SMCs in the tissue as agents. They have two rulesets that act on distinct timescales. First, there is the fast mechanical response that we model with a physical ruleset governing the cell repulsion and adhesion. It is used to simulate the structural dynamics of the vessel wall. The cells are modelled as spheres of a set radius which is based on the cell volume and governed by the biological solver. For the repulsion a Neo-Hookean potential is used, and for the attraction we use a linear force with a cutoff chosen such that only neighbouring cells can interact with each other:

(1) $$\left\{ \begin{array}{l} F=\;\frac{8{\text{a}}^{3}\text{C}(16{\text{a}}^{2}-36\pi \text{ aR}+27\pi^{2}\;{\text{R}}^{2})}{3\text{R}{\left(4\text{a}-3\pi \text{R}\right)}^{2}},\;\;d<{\text{R}}_{1}+{\text{R}}_{2}\\ F=\left(d-({\text{R}}_{1}+{\text{R}}_{2})\right)\cdot {\text{k}}_{\text{lin}},\;\;{\text{R}}_{1}+{\text{R}}_{2}<d<d_{\text{cutoff}}\\ \;F=0,\;\;d>d_{\text{cutoff}} \end{array}\right.$$

where a is the contact area and is approximated by

(2) $$\begin{array}{c} \text{a}=\sqrt{\frac{{\text{R}}_{1}{\text{R}}_{2}}{{\text{R}}_{1}+{\text{R}}_{2}}\cdot ({\text{R}}_{1}+{\text{R}}_{2}-\text{d})}, \end{array}$$

R_1_ and R_2_ are the radii of the SMCs, d is the distance between their centres, C is the elastic constant, k_lin_ is the linear force coefficient and d_cutoff_ is the force cutoff range. The forces act on the direction of the vector connecting the cells. We have also investigated other potentials and compared the resulting mechanical behaviour under stretching with experimental results, showing good agreement (Melnikova et al., in press). In future work we intend to test these more advanced potentials in the context of neointima formation.

Displacement of the cells is resolved using an over-damped Newton's law of motion, neglecting inertia:

(3) $$\gamma \frac{\text{d}\;\vec{{\text{r}}_{\text{i}}}}{\text{dt}}=\vec{{\text{F}}_{\text{i}}},$$

where γ is the damping coefficient, t is the time, and i is the particle index. A 4th-order Runge-Kutta method is used to integrate this law. This solver calculates vessel wall deformation during stent deployment and ensures that the cells stay in equilibrium during restenosis. The constants used in the physical model are detailed in Table 1.

**Table 1:** **Parameters of the mechanical model**.

**Parameter**	**Value**	**References**	**Comment**
*R*	0.015 mm	Tahir et al., 2014	For cells in the beginning of G0
*C*	0.1 MPa	Tahir et al., 2014	
*k*_*lin*_	0.01 N/mm	Tahir et al., 2014	
*d*_*cutoff*_	1.06 · (*R*_1_+*R*_2_)	Tahir et al., 2014	Value set to keep the cells from moving away from each other

The initial stent deployment is performed by radially expanding the stent from the centre of the vessel until it reaches a predetermined configuration. The stent is assumed to be much more rigid than the vessel wall, and the driving pressure in the balloon is assumed to be much higher than the counteracting pressure in the myocardium. Because of that the stent agents do not deviate from their radial trajectories as a result of interactions with vessel agents. Effectively, for the purposes of simulation, the stent agent masses are assumed to be infinite.

The second ruleset for the agents modelling SMCs is the biological solver. This submodel acts on the scale of hours to days and models cell growth, proliferation and death. The basic element of the biological solver is the cell cycle model (Tahir et al., 2014). It assumes that each cell is either in one of the states G1 (growth), S/G2/M (synthesis, secondary growth, and mitosis), or in the quiescent state G0. Over the course of G1 cells uniformly increase their volume to twice the initial value, unless their growth is interrupted. The interaction radius of the cell is adjusted accordingly. When a cell has spent a required amount of time in S/G2/M, it divides into two daughter cells, each with half the volume of the mother cell. The cell switches to S/G2/M if it is allowed to grow in G1 without interruptions for a set period of time. The criteria for growth inhibition are contact inhibition, which forbids growth if the entire neighbouring area of the cell is occupied, and nitric oxide (NO) inhibition, which is triggered if NO concentration in the cell rises above a threshold (Tahir et al., 2013). NO is produced by functional endothelial cells in response to wall shear stress (WSS). All cells are assumed to switch to the synthetic phenotype and enter G1 immediately after stenting, unless the contact inhibition (CI) criterion is fulfilled. If a cell is inhibited before or after it starts growing, it switches to the quiescent state G0, changes to contractile phenotype and does not start proliferating again even if the inhibition criterion becomes false on a later iteration. The contact inhibition parameters are selected so that the cell only starts growing if there is enough space for at least one new cell next to it, for example, if the cell in question is located next to a fenestration or a rupture of the IEL (Tahir et al., 2015). The parameters of this model are listed in Table 2.

**Table 2:** **Parameters of the biological model**.

**Parameter**	**Value**	**References**	**Comment**
*G*1 length	16 ± 2 h	Tahir et al., 2015	Normal distribution
S/G2/M length	16 h	Tahir et al., 2014, 2015	
Contact inhibition count	12		CI if the cells are densely packed
NO inhibition concentration	$0.001\frac{nmol}{mm^{3}}$	Coneski and Schoenfisch, 2012	

The internal elastic lamina (IEL) is modelled as a layer of agents similar to the SMCs on the inner surface of the artery wall. These agents have sizes and mechanical properties similar to the SMC agents, with one exception. When the lamina is stretched to more than 1.8 of its initial length (Holzapfel et al., 2005), it breaks and the over-stretched agent is removed. Before executing the restenotic growth simulation, fenestrations are generated in the IEL by switching a set percentage of the (randomly selected) IEL agents to SMCs (4%, Kwon et al., 1998). The percentage is selected to match the *in-vivo* percentage of the IEL surface occupied by fenestrations. A small sensitivity study for the contact inhibition number and the fenestration percentage can be found in the Supplementary Material. Changing IEL agents to SMCs instead of outright removing them was selected to account for the fact that in reality the IEL is very thin and there are no “deep holes” under the fenestrations.

**Figure 2:**
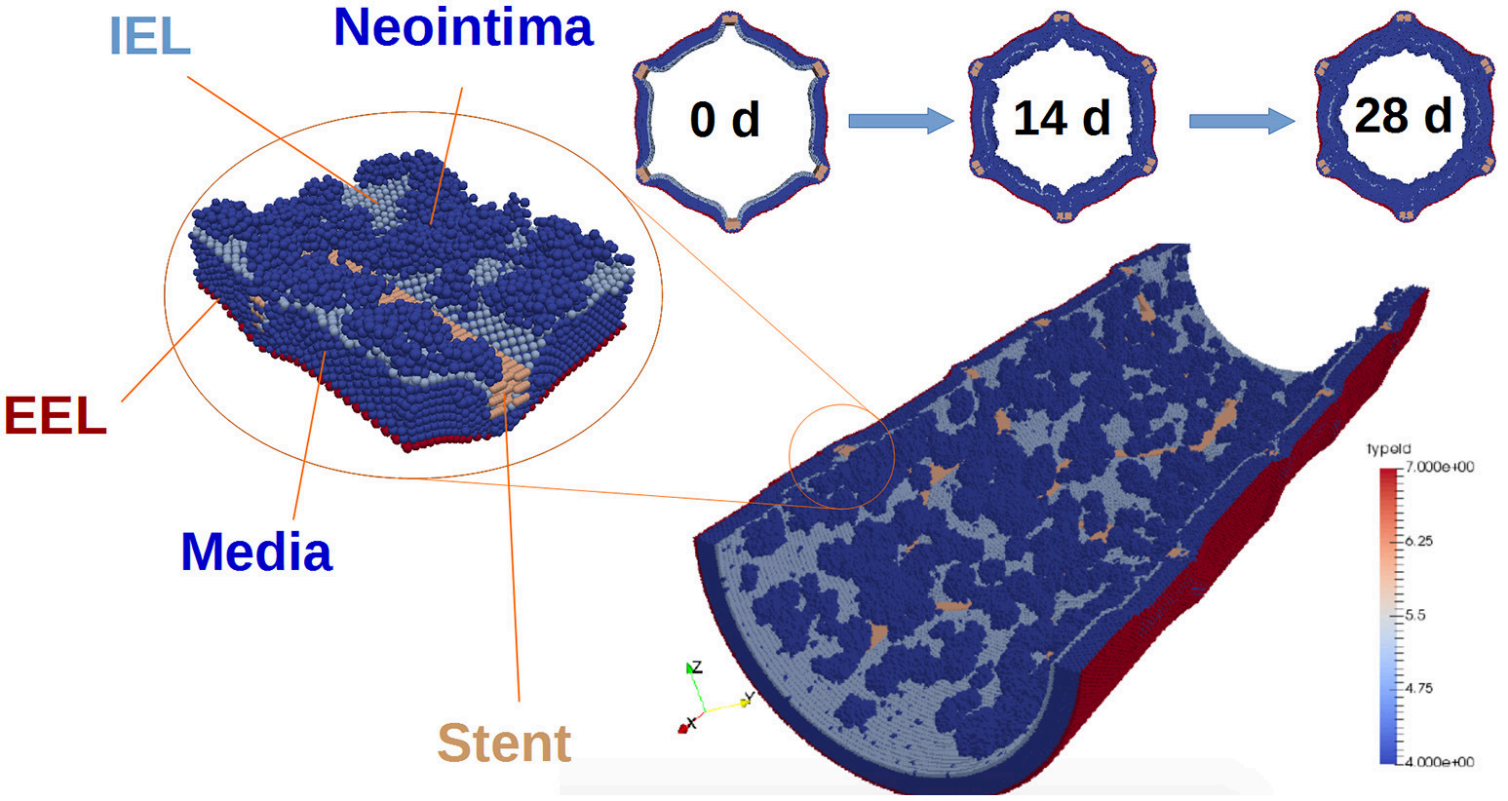
**Vessel wall model**. The figure illustrates restenosis in a porcine artery, which was injured by deploying a stent. SMCs are shown in dark blue, and stent agents in brown. External elastic lamina is red, and internal elastic lamina is light blue. Also, representative slices of the vessel wall at 0, 14, and 28 days after stenting are shown. Note that the blood flow is not shown in this picture.

The external elastic lamina (EEL) is modelled in a similar way to the IEL, with two notable exceptions. First, there are no fenestrations in the EEL. Second, during neointima formation (but not during stent deployment) the EEL agents are set to be immobile, since we assume that they are held in place by the adventitia. The vessel wall model is illustrated in Figure 2.

Another submodel is the blood flow model. For ISR3D, we model blood as an incompressible Newtonian fluid. This approximation is reasonable for the speeds and scales typical for stenosed vessels. The numerical solution is obtained by using a lattice Boltzmann model, implemented by Palabos (2013)^1^. For all blood flow calculations during the restenotic growth, the flux is kept constant. For our model we assume that neointima growth is governed by NO production, which is governed by the average wall shear stress (WSS), and because of that only the average flow through the vessel matters for the growth process. This flow is approximated by a steady flow. The Reynolds number *Re* is set to *Re*_0_ = 120 initially, typical for coronary arteries (Ku, 1997). As the restenosis develops, *Re* is decreased:

(4) $$Re_{new}=Re_{0}\cdot \;\sqrt{\frac{S_{0}}{S_{new}}\;}$$

**Figure 3:**
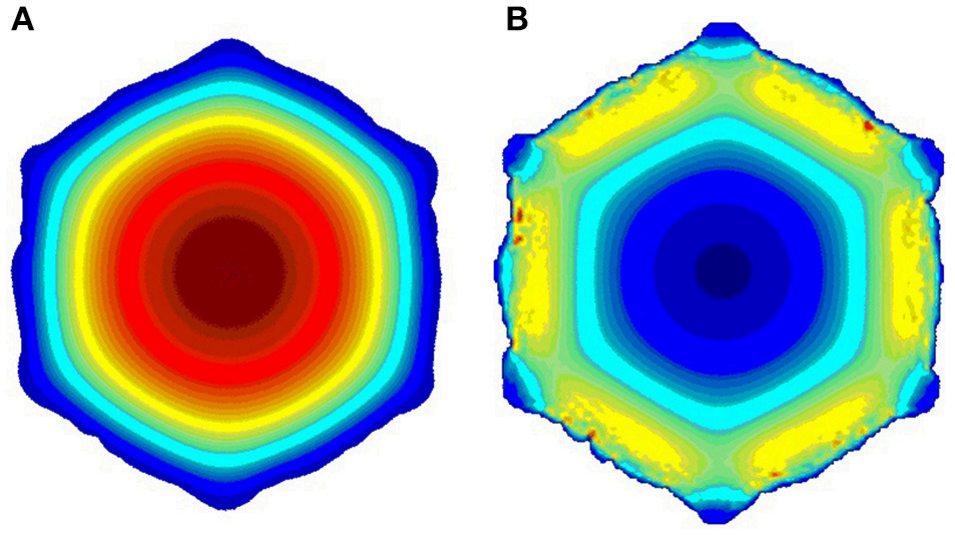
**Sample slices of the blood flow solution; (A)** velocity; **(B)** shear stress. (red is high, blue is low).

Here *Re*_*new*_ is the new Reynold number, *S*_0_ is the initial average vessel cross-section area, and *S*_*new*_ is the current average cross-section area. In this way we keep the blood flux constant as the restenosis develops, in accordance with the assumption that a constant blood flux is maintained to the myocardium. From the hydrodynamic solution, the WSS is obtained and passed to the biological solver. We use first order boundary conditions (bounce back), so the shear stresses are also first order of accuracy. In laminar flows, as we have, we still get sufficient accuracy, as demonstrated by Axner et al. (2009). Figure 3 shows sample velocity (a) and shear stress (b) profiles.

The agents that comprise the vessel wall cannot be used directly to form a no-slip boundary for the flow solver. Because of this, the cell agents are mapped to the same lattice used for the lattice Boltzmann solver, and the lattice cells that are at least partially occupied by SMCs are marked as solid. The resulting structure is smoothed and passed as a boundary condition for the flow. To pass WSS back to the cells, an opposite procedure is used: the stress is calculated for the lattice cells at the fluid-solid interface, and mapped to the SMCs that occupy them. For each surface SMC, WSS is averaged over the lattice cells occupied by that SMC. This value is used to calculate the amount of NO produced by the endothelial cells that cover this SMC, if they exist and are functional, as in Tahir et al. (2013, 2014).

We have performed numerical simulations using the model described above. The main parameters used in these simulations are presented in the Table 3. These parameters are the same for all results of the numeric experiments that are reported in Section Results. The flow lattice cell size was chosen after performing a test simulation with a finer grid (half the cell size used in the simulations presented here) and obtaining very close results (not shown).

**Table 3:** **Main numerical parameters of the simulation**.

**Parameter**	**Value**	**Comment**
Biological model timestep	1 h	
Flow lattice cell size	0.03125 mm	Typical SMC agent diameter
Blood velocity relative error	0.01	
Mechanical agent model convergence level	0.05	

To measure the neointima thickness at a time point we use the same procedure that is used in *in-vivo* experiments. We select several points on different stent struts and average the results (Gunn et al., 2002). Also, we qualitatively compare the overall shape of the simulated neointima to histology slices by taking representative images of the simulated tissue and comparing the shape and relative thickness of media and neointima to *in vivo* data.

## Results

### Stent deployment depth effect

Experimental studies associate extensive damage to the artery and related high injury score with increased neointima thickness and larger incidence of clinically recognised restenosis (Gunn et al., 2002; Morton et al., 2004; Iqbal et al., 2016). Injury is caused by stent malapposition, artery curvature, and high balloon-to-artery ratio. We aim to simulate the latter mechanism by regulating stent opening to achieve different injury scores (IS). IS 0 means that the stent is only touching the IEL; IS 1 means that the strut is pressed into IEL a bit, but the angle between IEL positions before and after stenting is less than 45°; IS 2 means that the angle is greater than 45°; IS 3 means that IEL is ruptured; and IS 4 means that the EEL is ruptured as well (Gunn et al., 2002). Because we do not model EEL rupture nor the supporting adventitial tissue, we do not simulate injury score 4 scenarios.

As an initial condition for our model, we use a stent geometry based on the BiodivYsio stent. This stent is inserted into the simulated vessel and expanded to different depths to cause different stretching and damage to the IEL and vessel wall in general. During this stretching, the strain of the IEL and stress on the SMCs are calculated. SMC agents are removed if the mechanical stress on them rises above a threshold, and IEL agents are removed if their strain is too high (see model descriptions in Section Methods and Model Description for details).

**Figure 4:**
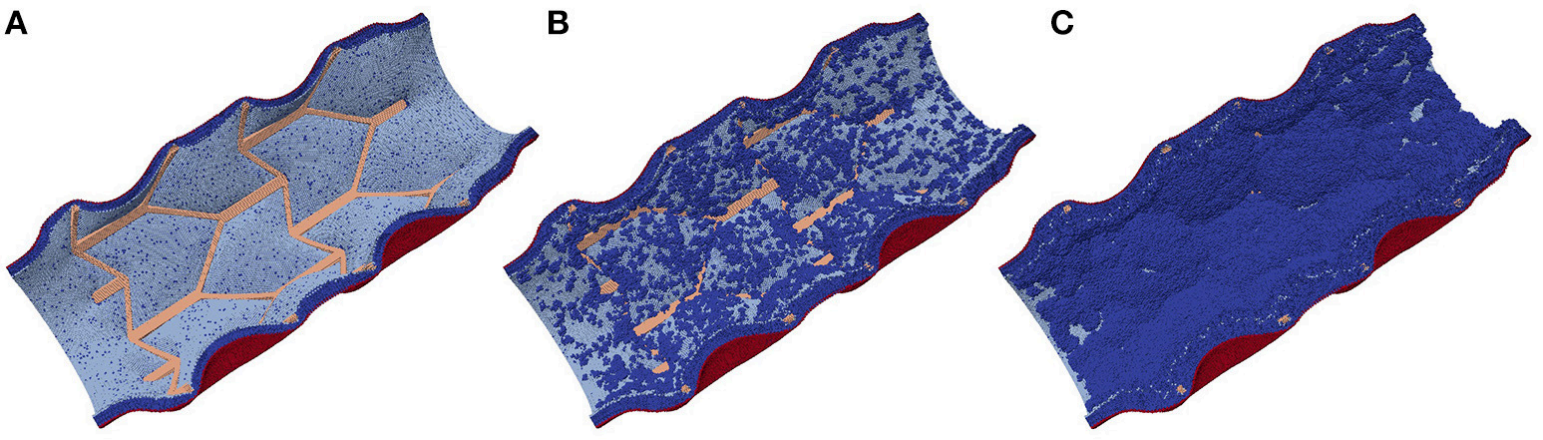
**Neointima proliferation for IS 3; (A)** vessel right after stenting; **(B)** 1 week after stenting; **(C)** 2 weeks after stenting.

After the initial deployment of the stent, the growth starts from two distinct parts of the vessel wall: first, SMCs become active at the locations ruptured by the stent, and second, some of the fenestrations in the IEL are stretched enough to allow migration of SMCs through them into the vessel lumen where they can start proliferating (Tahir et al., 2015; see Figure 4). The extent of this stretching, as well as the extent of the rupture, depends on the deployment depth.

**Figure 5:**
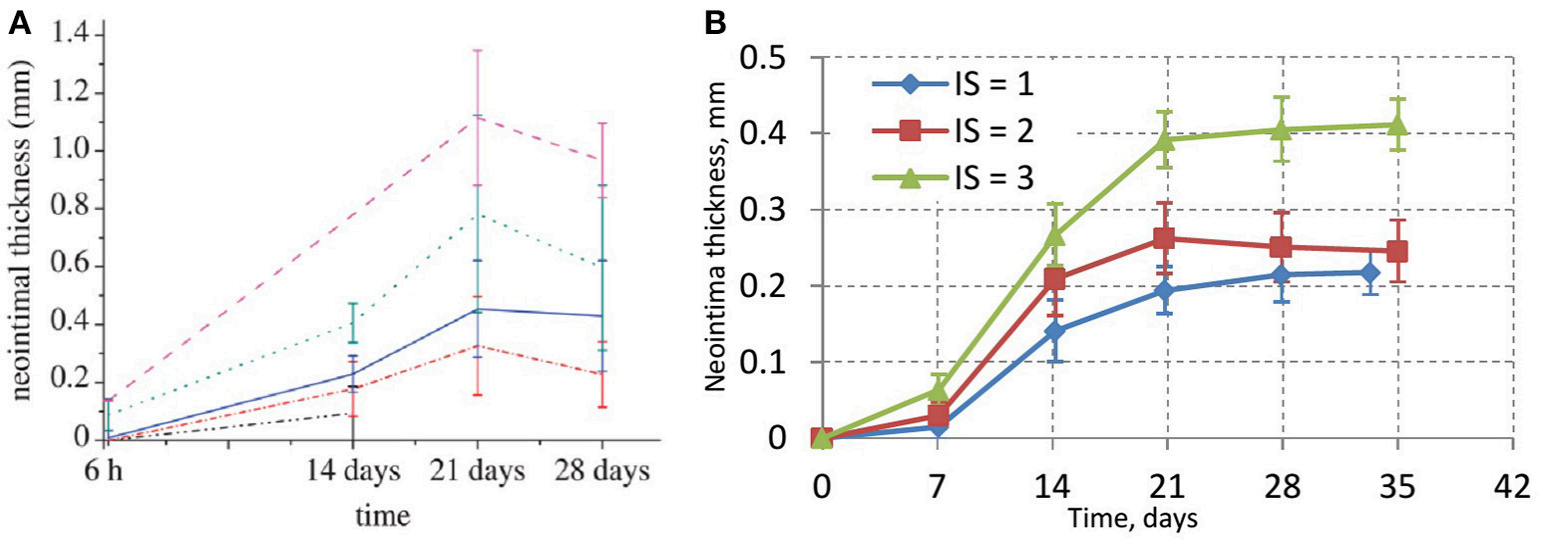
**(A)** *In-vivo* experimental data for neointimal growth. The mean and standard deviation of the neointimal measurements are plotted at 6 h, 14, 21, and 28 days post stent deployment. Black dashed-dotted line, injury score 0; red dashed-dotted line, injury score 1; blue solid line, injury score 2; green dotted line, injury score 3; pink dashed line, injury score 4 (Tahir et al., 2011); **(B)** simulated values of neointimal thickness.

The experimental porcine data is shown in Figure 5A (taken from Tahir et al., 2011). Figure 5B shows the results of different deployment scenarios for the same injury scores as in Figure 5A. For all these simulations a 15-day reendothelization scenario was assumed, as in Tahir et al. (2013, 2014), and based on experimental data (Nakazawa et al., 2010). The simulated growth curves qualitatively agree with the experimental ones, although quantitatively the simulated neointima thickness is significantly lower than in the real arteries. This is probably tied to the fact that real neointima is largely composed of extracellular matrix (ECM), while our current model does not take the ECM volume into account. After 15 days, the growth on the liquid-solid interface stops almost completely due to WSS being well over the threshold for a smoothly healed vessel, but there is still some residual growth inside the bulk of the neointima over the next few days. The initial neointima is not densely packed, so in some regions there is enough space for a cell to continue growing and dividing. The slight downward trend for IS2 could be explained by the compactification of neointima when the growth has almost stopped and the attraction force causes the cells to move closer to the equilibrium state.

### Reendothelization speed effect

Another factor that is associated with restenosis is the reendothelization speed (Van Beusekom et al., 2012). Functional endothelium suppresses the neointima formation by producing chemical factors under the influence of shear stress. There is an on-going effort to develop stents that cause a faster endothelium recovery, for example by covering them with progenitor cells, biologically relevant proteins, or by adding nanoparticles that bind to endothelial cells and provoke growth (Iqbal et al., 2013).

Because of that, we have studied the effect of reendothelization speed on neointima formation in our 3D model. Based on the experimental results and our model used in an earlier 2D model of ISR (Tahir et al., 2013, 2014), we have chosen three reendothelization scenarios: *normal*, with completely functional endothelium 15 days after stenting; *slow*, where endothelium recovers after 20 days; and *fast*, where reendothelization is complete in 10 days. For all these scenarios, we assume that 59% of the endothelial cells are functional after 3 days, and then the amount of functional cells grows linearly until it is 100% at the end point. New active cells are placed randomly, based on the study by Tahir et al. (2014). We use a 3D version of the endothelium model which is described in detail in Tahir et al. (2013, 2014).

**Figure 6:**
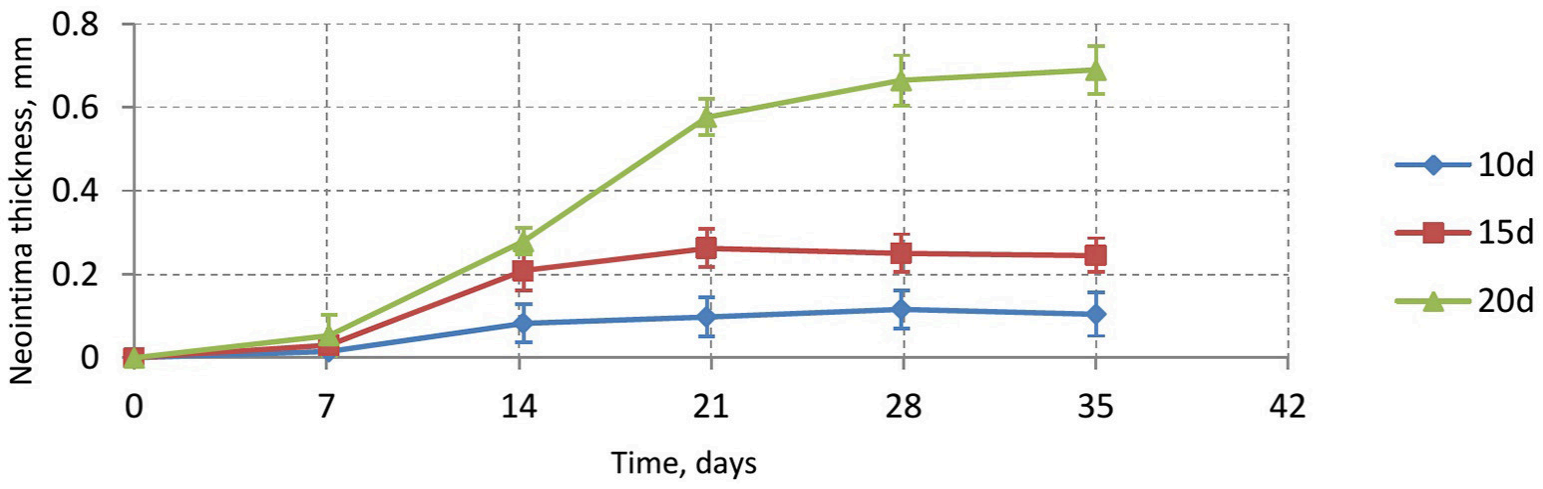
**Neointimal thickness dynamics for injury score 2 for different reendothelization speeds**.

**Figure 7:**
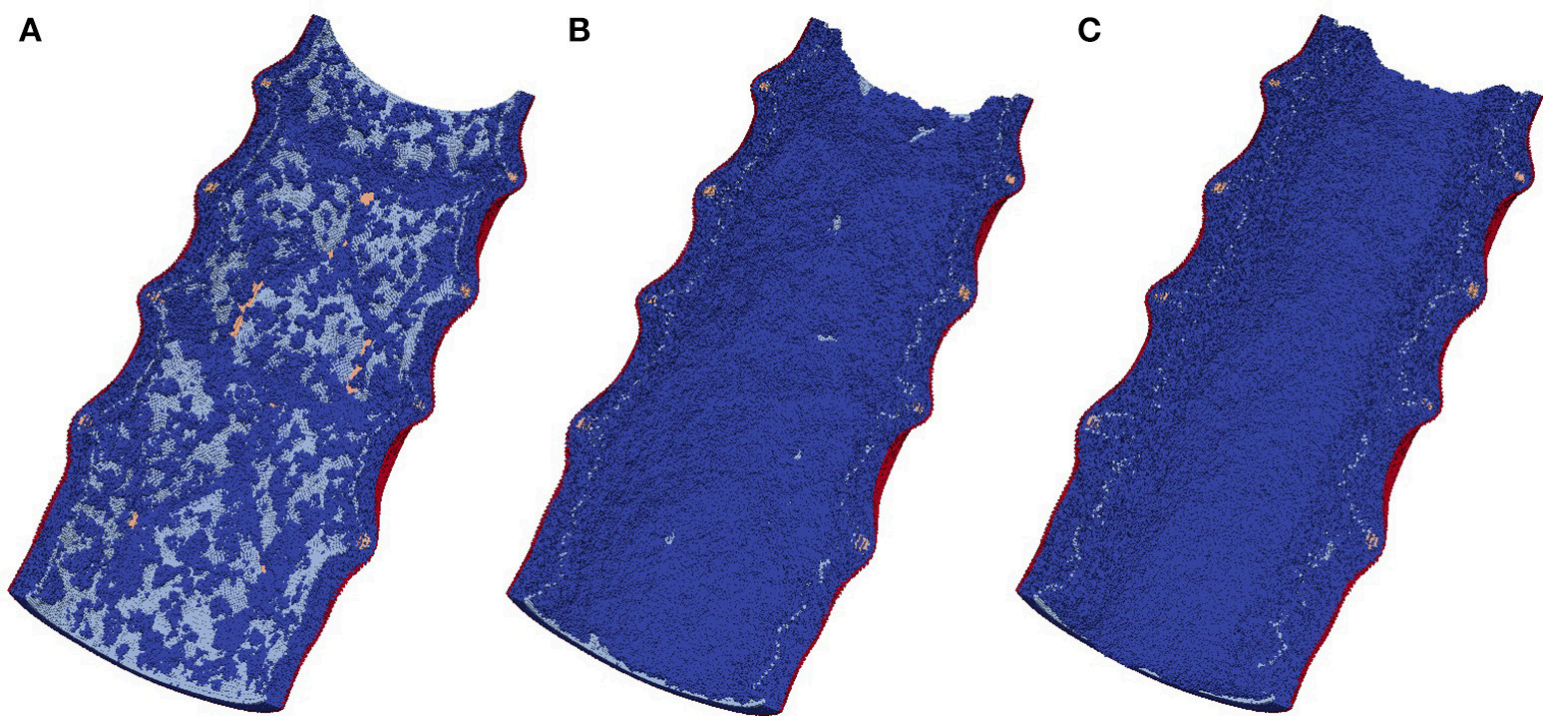
**Endpoints (35 days after stenting) for three reendothelization scenarios; (A)** 10-days scenario, gaps in the neointima are visible; **(B)** 15-days scenario, normally healed vessel; **(C)** 20-days scenario, restenotic vessel. See also growth dynamics in Figure 5.

Figure 6 shows the time dependence of the average neointima thickness for the reendothelization scenarios described above. For this study a vessel with IS 2 was selected, since this is a typical injury score in *in-vivo* studies (Gunn et al., 2002; Morton et al., 2004; Iqbal et al., 2016). The time course of reendothelization severely affects both the maximal neointimal thickness and the time when this thickness is reached. For the fastest scenario, there are gaps in the neointima and some stent struts are uncovered. Figure 7 shows the endpoints (35 days after stenting) for these three scenarios.

Our model is rather sensitive to this parameter, and relatively small changes in reendothelization speed can affect the final neointima thickness a lot. For the three scenarios described here, the average neointimal thickness over struts was equal to 0.10, 0.24, and 0.69 mm, for 10, 15, and 20 days scenarios, respectively. Note that the second case would be classified as a well healed lumen, whereas the third case is an actual restenosis (50% diameter occlusion) that would require additional treatment.

### Comparing NPAGF to *in-vivo* data

**Figure 8:**
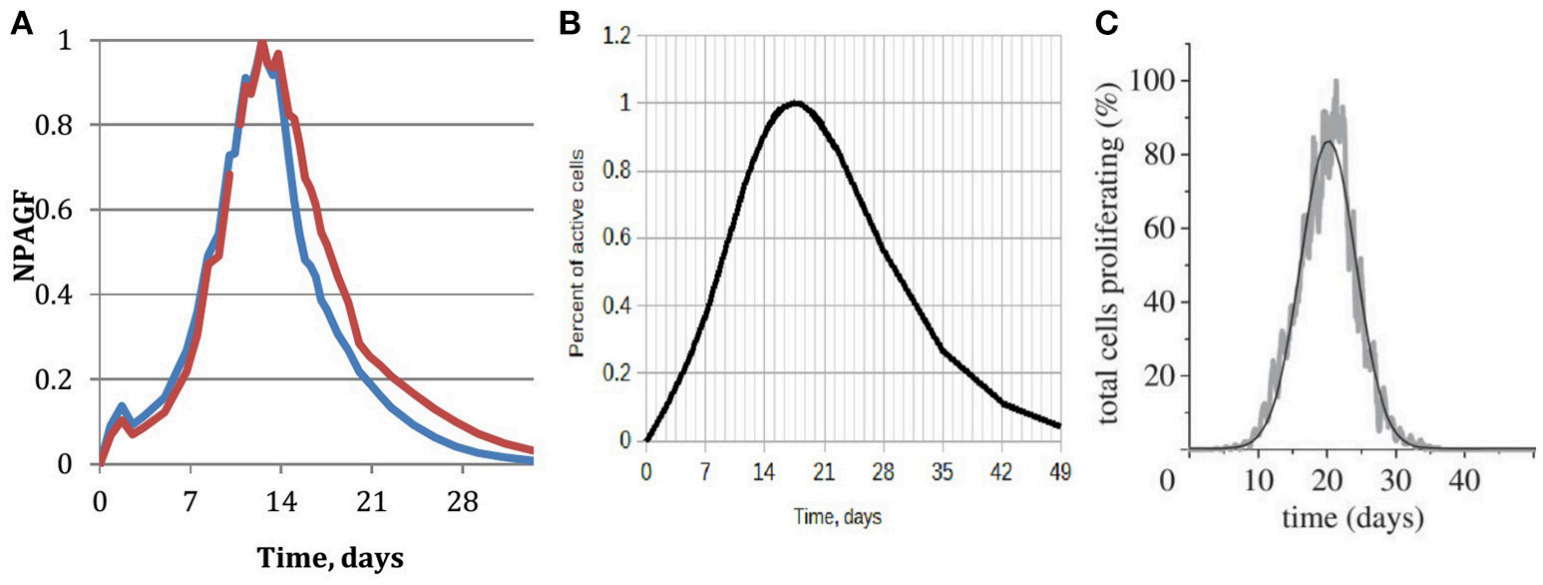
**Normalized peak absolute growth fraction. (A)** Obtained from the 3d simulation described in this paper. Blue—15 days reendothelization scenario, red—20 days; **(B)** analytical, fitted to experimental data, reproduced based on the data and equation from Schwartz et al. (1996); **(C)** obtained from our earlier 2D simulation, full reendothelization after 23 days (Tahir et al., 2014).

An important parameter of neointimal growth is the Normalized Peak Absolute Growth Fraction (NPAGF) (Schwartz et al., 1996). NPAGF is a metric for the total number of proliferating cells, and equals the product of growth fraction and the total cell number divided by the maximum value in the series. The time at which maximum growth occurs strongly depends on the species, with lower times for smaller species. A comparison between our simulated NPAGF and an analytical curve fitted to experimental data is shown in Figures 8A,B. The early peak in the simulated NPAGF in Figure 8A is caused by our assumption that all synthetic cells are at the beginning of their cell cycles immediately after stenting (they leave the quiescent state G0 and start proliferating), so the cell cycles are synchronized. Figure 8C shows NPAGF for the earlier 2D version of our model. The current 3D simulation has a much closer resemblance to the experiment-based curve, in particular it is asymmetric with a long “tail” of proliferating cells after most of the growth stops, while in the 2D simulation the function is symmetrical (even Gaussian shaped, see Tahir et al., 2014). Also, in the 3D case, as well as in the experiment-based case, a notable growth starts immediately after stenting, while in 2D case the growth during the first week is almost negligible.

It should be stressed that the only difference between the 2D and 3D models that is not related to the dimensionality of the models is the presence of fenestrations in 3D. According to our model, the fenestrations are active during the early stages of restenosis, before a continuous neointima forms. Higher concentration of fenestrations leads to more initial growth locations on the IEL away from the stent struts and results in faster early growth and a thicker neointima. The late growth, on the other hand, is completely determined by the difference in 2D and 3D geometry: in the latter case more cells inside the neointima have enough space to grow and proliferate.

### Effects of stent strut width on neointima growth

**Figure 9:**
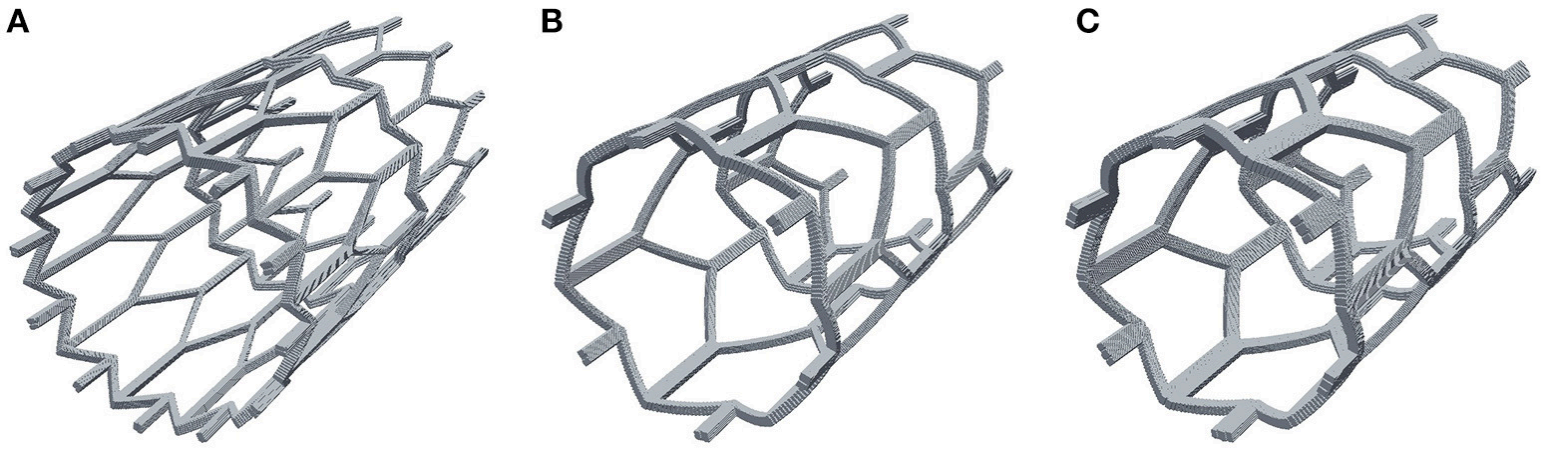
**Stent configurations used to study the effect of stent width on neointimal growth. (A)** Narrow struts, width w = 0.1 mm; **(B)** normal struts, w = 0.2 mm; **(C)** wide struts, w = 0.3 mm.

It is well known that to optimize treatment outcome, the amount of exposed stent struts has to be minimized, and that stents with bigger struts cause more damage to the vessel and have a greater risk to cause a restenosis (Briguori et al., 2002). In this section we present the results of comparing different stent configurations (Figure 9). The default model that was used in the previous sections (strut width *w* = 0.2 mm, Figure 9B), which we will compare to a model with wider struts (*w* = 0.3 mm, Figure 9C), and a model with an increased number of smaller struts (*w* = 0.1 mm, Figure 9A). Again, for this comparison we use the 15-day reendothelization scenario and IS 2, as these are the parameters that approximate the *in-vivo* growth and experimental conditions the best.

**Figure 10:**
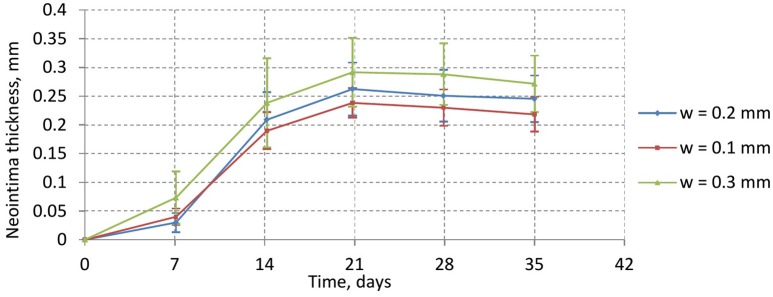
**average neointima thickness over struts for normal (w = 0.2 mm), wide (w = 0.3 mm), and narrow (w = 0.1 mm) struts**.

Figure 10 shows the time dynamics of the average neointima thickness over strut for the stent designs described above. In the current model the strut width only has a minor effect on neointima formation. The reason for this might be the absence of ECM in our model. ECM fills the gaps in neointima and increases its volume. The only factor we account for in our model is that wider stents cause more damage to the vessel wall and stretch neointima more. However, for the deployment depth we have used in this simulation, the extent of this damage is relatively small for all configurations used, so the effect on the growth dynamics is not very pronounced. We do however observe a positive correlation between strut width and neointimal growth.

### Simulating a real-sized stent

**Figure 11:**
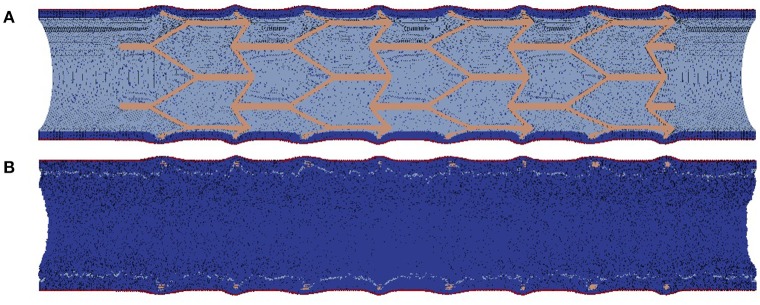
**real-sized stent simulation; (A)** right after stenting; **(B)** 35 days after stenting. The growth happens outside the stented area because we assume that the endothelium is denudated in the entire simulated segment.

To demonstrate that our simulation framework can be used to simulate restenosis for realistic geometries, we have simulated 1 month of restenosis in a real-sized vessel. For this proof-of-concept simulation we have used a stent geometry composed of four sections of the BiodYvisio stent, in total 16 mm in length. This stent was deployed in an 18 mm long, 2.8 mm wide vessel. The start and end points for this simulation are shown in Figure 11. Note that for this simulation we have tracked the behaviour of more than 5 million individual smooth muscle cells, over 870 biological timesteps of 1 h, while fully coupled to the blood flow simulator. For such simulations we rely on large scale computing capabilities (see e.g., Groen et al., 2013; Borgdorff et al., 2014).

The stent in Figure 11 is two times longer than those in the other simulations described above, and is deployed to a similarly-sized vessel with similar flow. As a result, the growth dynamics in the stented area is similar to that of the shorter vessel, and so, there is not much sense to simulate such a large segment. However, in a real vessel the tortuosity of the vessel and the edge effects of stent deployment (different damage to the IEL and the endothelium, for example), can affect restenotic growth (Duraiswamy et al., 2007; Amatruda et al., 2014). To capture these effects the whole stent has to be simulated. Here we demonstrate that our modelling framework is currently capable of simulating such systems, and in the future we intend to look in more detail to effects of curvature and edge effects.

## Discussion

In this study we describe our multiscale 3D model for in-stent restenosis and report first results obtained using this model. We perform simulations in which we vary parameters that are thought to be responsible for altering the restenosis dynamics. The computational experiments demonstrate that the effects observed *in vivo* are present in our model as well, although sometimes to a smaller extent. It is likely that these discrepancies can be alleviated by tuning the model parameters or by including explicit extracellular matrix into the model of the vessel wall.

Going into more detail, our simulated response as function of stent deployment depth is close to the *in vivo* data for IS 1, about 1.5 times smaller than *in vivo* for IS 2, and almost 2 times smaller for IS 3. The reason for this may lie in the extracellular matrix, which forms a large part of neointima *in vivo* (Farb et al., 2004).

Reendothelization speed affects the process as expected from *in vivo* data, however, it is hard to obtain exact *in vivo* results for the growth dynamics of functional endothelium (Van Beusekom et al., 2012). Given the sensitivity of our model for this parameter, and results obtained in our earlier 2D studies (Tahir et al., 2013, 2014), we would suggest to study in some more detail these reendothelialization. It is worth mentioning that for abnormal endothelial regeneration speed we obtain not just a restenotic scenario for slower regeneration, but also an incomplete coverage scenario for faster regeneration. *In vivo* this scenario would mean increased thrombogenicity of this stent, increasing the risk of late thrombosis.

NPAGF is close to the analytical function fitted to the experimental data for the 1st month after stenting, but after that the simulated and analytical functions diverge slightly. This can be caused by two different reasons. First, the model might not capture features of late remodelling due to absence of ECM. Second, the analytical function, fitted to the actual experimental data, itself might not be entirely accurate. It has good agreement with experimental data for the 1st month after stenting, but after that there are only a few datapoints (14, 28, and 168 days) and the function fits them imperfectly: it fits the 14 days datapoint, but it underestimates the 28 days one and overestimates the 168 days point. The data points suggest late downward remodelling, while the fit function remains constant for that period (Schwartz et al., 1996).

Different stent widths affect the growth only a little, and from *in-vivo* data one could expect a more pronounced effect. This might be because in reality wider stent struts affect the increase in neointima through mechanisms other than pure damage to the vessel wall, for example by increasing thrombogenicity or by accumulating more ECM on their surface.

Finally, we demonstrate that our modelling framework can be used in the future for simulating neointimal growth in realistic stent geometries, obtained for example using microCT data as in Amatruda et al. (2014).

There are several model parameters that can be tuned to affect growth dynamics. First of all, we approximate the elongated SMCs as round agents, with a radius selected to match the real SMCs by volume. This is a very rough approximation, and the cell size affects the dynamics a lot. Simply by tuning the radius of the model agent we can affect the volumetric growth speed of the tissue. During each cell cycle the volume of the tissue is increased by an amount equal to the initial volume of the cells that divided during this cycle. Since most of the growth happens at the fluid-solid interface, this volume is close to the volume of a single surface layer, which depends on the radius of a single cell. Also, the radius (and shape) of the SMCs affect their interaction with the stent struts, the mechanical stresses, and consequently the extent of damage they suffer when the stent is deployed and the healing response.

Another parameter that directly affects the growth rate is the duration of a single cell cycle. However, there is a lot of experimental data for that parameter, which we use to select an appropriate value for our model.

However, while the above-mentioned parameters are based on experimental values, there are also purely synthetic parameters in the model. The contact inhibition criterion is based on the number of cells in the neighbourhood of the cell. We select 12 cells as the inhibition value for SMCs, based on the assumption that the cells are arranged closely to dense packing and that the cell continues growing unless its whole neighbourhood is occupied. Also, the size of this neighbourhood is ultimately arbitrary, selected to be “slightly larger” (in fact, 6% larger) than the sum of the radii of the two interacting cells. Although this inhibition is backed up by biological results (Evans et al., 2008), we need to include more accurate modelling of SMC biology (Parton et al., 2016), for example, mention some recent models for SMC growth), including the response of SMC to transmural pressure (DeMaio et al., 2004).

The exact nature of the cell-cell mechanical interaction force can also affect the vessel response to injury. We use a relatively straightforward interaction potential and also, for example, do not model the collagen and elastin molecules that are part of the ECM and are responsible for the mechanical properties of the artery when it is severely stretched. We do not include these molecules in our model since we assume that during stent deployment the artery is not stretched to such an extreme extent. However, there are vessel wall models that account for these molecules (Witthoft et al., 2016).

Another notable aspect of the ECM that we ignore is its volume. In the tunica media, this volume is negligible, but there are studies that suggest that a large part of the neointima consists of ECM, although there are many different estimates of the exact percentage of ECM in neointima (see for example Farb et al., 2004). Also, ECM can trap water during the early stages of restenosis, which causes swelling in the first few weeks after stenting and shrinkage at the later time when the water is released back into the bloodstream (Kim et al., 2000).

In the current version of the model, the growth dynamics agrees best with the experimental results for the 15-day reendothelization scenario, producing a mostly properly healed vessel. However, a truly restenotic vessel is produced only in the 20-day scenario, although in this case the growth later than we expect from experiments.

Overall, the 3D ISR model shows improved results compared to the earlier 2D model, but it clearly requires additional extensions and validation.

## Conclusion

In this paper we have presented the 3D fully-coupled multiscale model of restenosis and the results of testing it for different sets of parameters to see oh varying the model parameters and initial conditions affects the restenotic growth. For the parameters based on experimental results, the results are in qualitative agreement with *in-vivo* data. Neointima thickness dynamics qualitatively agrees with experiment except for late remodelling; quantitative differences are likely due to extracellular matrix (ECM), which is not yet included in the model.

In the future we plan to add ECM to the model, since it sometimes makes up more than 50% of the lesion. As one of the next steps we also plan to find more experimental pig data for NI thickness to validate the model, since currently only a relatively small dataset is available. Then we plan to test the model on realistic vessel geometries (3D porcine coronary vessel models built from MRI scans) and after validation to transition from porcine to human physiology.

## Author contributions

PZ implemented the current version of the model, designed, and performed the simulations for variable injury score and variable reendothelization speed, analysed the results and drafted the manuscript. TA designed, performed, and analysed the simulation of variable stent designs. AS contributed to the mechanical model and helped analyse the data. AH conceived of the study, designed the study, coordinated the study, and helped draft the manuscript. All authors gave final approval for publication.

## Funding

We acknowledge financial support by The Russian Science Foundation, Agreement #14-11-00826 (10.07.2014). We also acknowledge partial funding from the European Union Horizon 2020 research and innovation programme under grant agreement no. 671564, the ComPat project (http://www.compat-project.eu/) and under grant agreement 675451, the CompBioMed project (http://www.compbiomed.eu/).

### Conflict of interest statement

The authors declare that the research was conducted in the absence of any commercial or financial relationships that could be construed as a potential conflict of interest.

## Footnotes

^1^http://www.palabos.org.
