# Supplementary material for: A Comparison of Fully-Coupled 3D In-Stent Restenosis Simulations to In-vivo Data
Source: Front Physiol. 2017 May 23;8:284. doi: 10.3389/fphys.2017.00284 (PMC5440556; doi:10.3389/fphys.2017.00284)
Supplement: Supplementary file 1 [file DataSheet1.DOCX]

Supplementary Material

A comparison of fully-coupled 3D in-stent restenosis simulations to in-vivo data

Pavel Zun^1,2*^, Tatiana Anikina^1,2^, Andrew Svitenkov^1^, Alfons G. Hoekstra^1,2*^

^1^ITMO University, St. Petersburg, Russia

^2^Computational Science Lab, Institute for Informatics, Faculty of Science, University of Amsterdam, The Netherlands

*** Correspondence:**
Pavel Zun [pavel.zun@gmail.com](mailto:pavel.zun@gmail.com)

Alfons G. Hoesktra [a.g.hoekstra@uva.nl](mailto:a.g.hoekstra@uva.nl)

# Sensitivity study

The model described in the main article contains a multitude of parameters, most of which are selected based on experimental data. The most notable of these parameters is the endothelium regeneration speed, the effects of which are described in the main article in section 3.2. To study the variance of other important parameters on the growth process, we have performed a simulation using the replica computing pattern. The parameters which were tested in this experiment are the percentage of pores (fenestrations) in the internal membrane and the threshold weight of the cell’s neighbours *CI*, exceeding which stops the cell growth. The pore percentage is based upon the data about the concentration and the size of pores in experimental samples, and the *CI* is selected based on the cell configuration in the model and on the experimental end configuration.

Figure 1 shows the results of modelling for different pairs of these parameters. The cell growth is estimated based on the amount of new cells in the vessel lumen. Typical stent deployment parameters are used: strut width of 0.2 mm and injury score (IS) 2, i.e. the membrane is stretched but not ruptured. The data for the fenestration percentage of 0.06 and CI = 6.5 is absent due to a technical error during the simulation.

The results show that for the pore percentage, the biggest difference in growth shows up at the moment of qualitative transition from the absence of pores to their presence, while increasing the number of pores has a much smaller impact. Without the pores, the cells can only move to the lumen through the ruptures in the IEL (which aren’t present in this deployment configuration), so there is almost no growth for CI 6.5 and 8.5 (12 new cells for CI = 6.5 and 8025 for CI = 8.5). For CI = 10.5 a minor growth inside the vessel wall is observed. In the presence of the fenestrations, the CI value is much more important than the exact percentage of the fenestrations. For further modelling, a value of 4% fenestrations and CI = 10.5 were chosen, since they agree with the experiment the best.


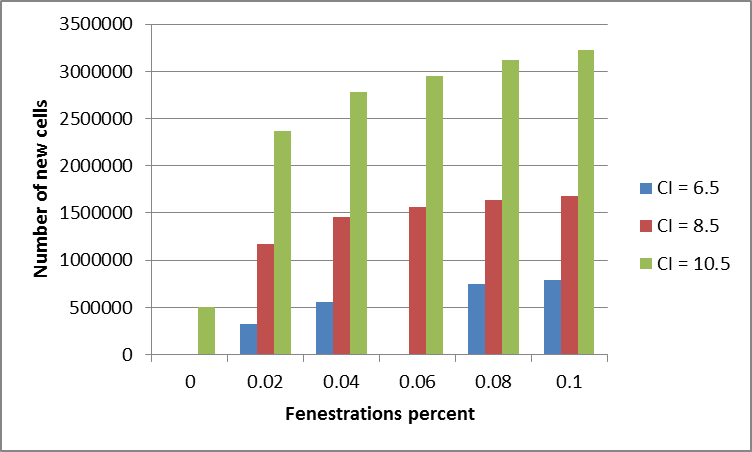


**Supplementary Figure 1.** 35 day endpoint number of new cells for different modelling parameters. No data for parameters (0.06, 6.5).
